# The paediatric participation scale measuring participation restrictions among former Buruli Ulcer patients under the age of 15 in Ghana and Benin: Development and first validation results

**DOI:** 10.1371/journal.pntd.0007273

**Published:** 2019-03-14

**Authors:** Dorien T. Beeres, Jacolien Horstman, Pierre van der Tak, Richard O. Phillips, Kabiru M. Abass, Tjip van der Werf, Roch C. Johnson, Ghislain E. Sopoh, Janine de Zeeuw, Pieter U. Dijkstra, Yves T. Barogui, Ymkje Stienstra

**Affiliations:** 1 University of Groningen, Department of Internal Medicine/Infectious Diseases, University Medical Centre Groningen, Groningen, Netherlands; 2 Kwame Nkrumah University of Science and Technology (KNUST), School of Medical Sciences and Kumasi Centre for Collaborative Research in Tropical Medicine (KCCR), Kumasi, Ghana; 3 Agogo Presbyterian Hospital, Agogo, Ghana; 4 Centre Interfacultaire de Formation et de Recherche en Environnement pour le Développement Durable, Université d'Abomey-Calavi, Abomey-Calavi, Benin; 5 Programme National Lutte contre la Lèpre et l'Ulcère de Buruli, Ministère de la Santé, Cotonou, Benin; 6 Department of Rehabilitation Medicine, Center for Rehabilitation, and Department of Oral and Maxillofacial surgery, University Medical Center Groningen, University of Groningen,The Netherlands; University of Tennessee, UNITED STATES

## Abstract

**Background:**

Buruli Ulcer (BU) is a neglected tropical disease caused by *Mycobacterium ulcerans*. Former BU patients may experience participation restrictions due to physical limitations, stigmatization and other social factors. A scale that measures participation restrictions among children, who represent almost half of the affected population, has not been developed yet. Here, we present the development of a scale that measures participation restrictions in former BU paediatric patients, the psychometric properties of this scale and the scales’ results.

**Methods:**

Items were selected and a scale was developed based on interviews with health care workers and former BU patients in and around the BU treatment centre in Lalo, Benin. Construct validity was tested using six a priori formulated hypotheses. Former BU patients under 15 years of age who received treatment in one of the BU treatment centres in Ghana and Benin between 2007–2012 were interviewed.

**Results:**

A feasible 16-item scale that measures the concept of participation among children under 15 years of age was developed. In total, 109 (Ghana) and 90 (Benin) former BU patients were interviewed between 2012–2017. Five construct validity hypotheses were confirmed of which 2 hypotheses related to associations with existing questionnaires were statistically significant (p<0.05).

In Ghana 77% of the former patients had a Paediatric Participation (PP) scale score of 0 compared to 22% in Benin. More severe lesions related to BU were seen in Benin. Most of the reported participation problems were related to sports, mainly in playing games with others, going to the playfield and doing sports at school.

**Conclusion:**

The preliminary results of the PP-scale validation are promising but further validation is needed. The developed PP-scale may be valid for use in patients with more severe BU lesions. This is the first research to confirm that former BU patients under 15-year face participation restrictions in important aspects of their lives.

## Author summary

Buruli Ulcer is a neglected tropical disease caused by infection with *Mycobacterium ulcerans*. While treatment has largely improved, former BU patients may experience participation restrictions after treatment, due to physical limitations, stigmatization or other social factors.

With more than half of the BU patient population in Africa being children, a scale that measures participation restrictions among former paediatric BU patients is relevant but has not been developed yet. Here, we present a scale that measures participation restrictions among former BU patients under the age of 15 year that is suitable for low-income settings. The 16-item scale was developed in Benin and tested in both Ghana and Benin. The scale shows good performance and may be used for several diseases that occur in similar settings and result in disabilities (both physical and/or social).

## Introduction

Buruli Ulcer (BU), caused by infection with *Mycobacterium ulcerans (M*. *ulcerans)* is a neglected tropical disease that has been reported in 33 countries, predominantly tropical and subtropical regions, but is mostly prevalent in sub-Saharan countries [1]. In 2016, almost 80% of all new BU cases were reported in West Africa, mainly Côte d’Ivoire, Ghana, Benin, and Nigeria [2]. Recently, an increase in both the spread of BU cases and the number of severe cases in South-eastern Australia has been noticed [3]. Compared to the population distribution of BU cases in West-Africa, where 48% of BU patients are under 15 years of age, in that study the median age was 58 years (IQR 38;74), and only 10% was under the age of 15 years [3].

*M*. *ulcerans* infection produces a toxin that leads to destruction of the skin and soft tissue. The proportion of affected body parts and categories of disease differ per geographical region. In the region of Western Africa, the limbs are the most frequently affected body parts with 55% of lesions on the lower limbs, 35% on the upper limbs and only 10% on other parts of the body [4]. Treatment consists of wound care and antibiotic treatment with rifampicin/streptomycin or rifampicin/clarithromycin [5]. Surgery was once the mainstay of treatment of BU, but a recently published trial showed that even large ulcers can heal with antibiotics and wound care alone [6]. BU lesions are categorized based on severity: Category I for single small lesions <5cm in diameter, Category II for single non-ulcerative and ulcerative plaque and oedematous lesions with a diameter of 5-15cm, and category III for lesions >15 cm in diameter, multiple lesions, lesions at critical sites as head and neck region and genital region, disseminated and mixed forms as osteitis, osteomyelitis and joint involvement [5].

Although the mortality from BU is low, it frequently leads to disability such as restriction in range of motion of joints contributing to functional limitations [7]. Apart from functional limitations, stigmatization and economic burden may impact social life of (former) patients, such as school dropout and unemployment [8,9]. A qualitative study showed a high impact of BU on social life and maintenance of work/school, and a need among former patients with BU for support and counselling on how to deal with economic and social burden as a result of BU [10].

To measure the impact of disease on an individual and their environment, the WHO established the International Classification of Function, Disability and Health (ICF) criteria that describe disabilities in terms of impairments, activity limitations and participation restrictions [9]. Participation restriction is defined as ‘any problem an individual may experience in involvement in nine life domains such as learning and self-care, domestic life, interpersonal interactions and relationship and social and civic life’ [9]. Based on this definition a participation scale (P-scale) was developed to measure perceived restrictions in these life situations among adult individuals with disabilities in low and middle-income countries [11]. The P-scale consists of 18 items and has been used to measure participation restrictions among former patients with BU, leprosy, poliomyelitis or spinal cord injuries [11,12].

The P-scale among former adult BU patients demonstrated long term participation restrictions mainly among patients with large lesions [12,13]. To our knowledge, the P-scale has only been used in adults. In sub-Saharan Africa, almost half of the BU patients are children under the age of 15 years, but a scale to evaluate participation problems in this group does not exist [1,5]. This study focuses on the development of a participation scale suitable for former BU patients under 15 years of age (Paediatric Participation scale: PP-scale).

The aim of this study is to develop a scale to measure participation problems in children after treatment for BU, to analyse the psychometric properties of this scale as used in Ghana and Benin and to present scale results.

## Methods

Requirements were set for different participation domains that needed to be measured, as described by the ICF [9]. The development process consisted of three phases;

Phase I: Item collection and formulation of first draft questionnaire;

Phase II: Item reduction analysis and content validity of the draft scale;

Phase III: Psychometric testing and analysis [10].

### Phase I: Item collection and formulation of first draft questionnaire

#### Study participants

For the first phase of the study medical specialists, teachers, other community workers, chiefs and parents of former patients were included and interviewed for item collection at the BU Treatment Centre in Lalo, Benin and in its surrounding villages based on purposive sampling.

#### Scale development process

During the interviews, the participants were asked to imagine a boy/girl aged 3 years living in the village and answer five sections of questions related to their participation.

In the first section, daily activities were questioned, such as the estimated age a child starts eating independently, age when a child starts collecting water, age when a child participates in ceremonies, age the child starts with household responsibilities. Secondly, participants were asked to imagine a young boy (aged between 6–12) who is affected with BU and just finished treatment. Questions on their knowledge of the disease, cause of disease, treatment and consequences of BU were asked, as well as their thoughts on the way others in the community view (former) BU patients. In the third section, participants were again asked to imagine the same boy at school. Questions on the responsibilities and barriers the child may face at school were asked (e.g. if the disease would influence the child’s opportunity to participate in class, to participate in school-related activities, establish relationship with peers/teachers). In the fourth section, responsibilities of the child in household activities were questioned, as well as barriers the child may face as a result of the disease. In the last section, questions with regard to community participation were asked (e.g. if the child has similar opportunities as other children of the same age to participate in community events, what could be potential barriers, religious constrains etc). Based on the responses of the participants in these interviews, general practices and potential barriers with relation to BU were identified. Relevant items were collected and questions were formulated to be tested in phase II. The items were aimed to be understandable from the age of 5 year and therefore needed to be simple.

#### Peer comparison concept & response options

Roles and participation may differ between individuals within the community and it can be difficult for participants to compare situations before and after their disease. Therefore, Brakel et al (2006) used in the P-scale the ‘peer comparison concept’ to partly overcome these challenges [11]. For the PP-scale the ‘peer comparison concept’ was also used. Responders were asked to compare themselves with their peers, and items were formulated accordingly (e.g. *Can you go to the market just like the other children do*?*)*. The response options for the PP-scale are similar to the response options of the P-scale (*Yes/ Sometimes/ No)*. If the answer was ‘Sometimes’ or ‘No’, the responders were asked to rate the importance of this limitation using a 5-item Likert scale. To each response option a numeric value is attached to calculate the total sum score. The response options are: ‘Yes (0)’; ‘No problem (1)’ *(the participant does not experience a participation restriction*; ‘A little bit important (2)’ *(the participant experiences some participation restriction and considers it a medium important restriction)*; ‘Important (3)’ *(the participant has difficulties with the item and considers it an important restriction)*; ‘Very important (5)’ *(if the participant is not able to perform the item and considers it highly important)*. If the item was not relevant to the participant, the participant could answer with either ‘Irrelevant/I don’t want to/I don’t have to (0)’*(if the question is not relevant for the participant)* or ‘Not specified, not answered (0)’ *(if the question does not apply to the participant or was not answered*, *e*.*g*. *participant is too young for an activity)*. The total sum-score is calculated by adding up the scores of all individual items. The lowest PP-scale sum score possible is 0 and the maximum PP-scale sum score possible is 80, indicating the poorest score.

### Phase II: Item reduction analysis and content validity of the draft scale

#### Study participants

After collecting items of the paediatric participation scale, former patients under 15 years of age were included and invited to participate in the pre-testing of the collected items at the BU Treatment Centre in Lalo, Benin and in its surrounding villages based on convenience sampling. Information on socio-economic characteristics (e.g. village, religion, profession, type of housing, access to water, access to electricity, stable income, disease insurance. Information on the disease and treatment (date of diagnosis, duration of disease, type of treatment, category of lesion, treatment outcome, presence and position of BU related lesions) was retrieved from medical records.

#### Scale development process

First the understandability of the questions and response options were tested (Phase II.1) Secondly, items were tested for endorsement, discrimination ability and correlation with sociodemographic factors (Phase II.2). To test for endorsement, items with a positive response of less than 10% or over 90% were excluded. Items that showed a strong correlation with socio-demographic variables were excluded. After elimination of items, a draft questionnaire was composed to be tested in phase III.

### Phase III: Psychometric testing and analysis

#### Study participants

For the third phase former patients were identified using patients’ records kept by the BU health-care workers of the hospital/treatment centre in Agogo, Ghana and Allada/Lalo, Benin. In Ghana, the patients’ records proved to be incomplete. Therefore, sampling strategy was adjusted and convenience sampling at schools located within the reach of the hospital were performed. Community volunteers were asked to identify former patients at the schools.

In Benin, convenience sampling based on the accessibility of village of origin of the participant and the number of former patients living in the village was used. Criteria for inclusion as former patient was being diagnosed with BU (confirmed by PCR) in one of the treatment hospitals between 2007–2012, having finished treatment at least three months earlier and being below age of 15 years at the time of the interview. Exclusion criteria were not being able to give informed consent and being <5 years old. Participant characteristics, treatment (antibiotics/surgery), category of lesion, and joint involvement were collected from the medical records if available. Community controls were selected using frequency matching based on age (+5/-5 years, minimum age of 5 years), gender and geographical location. All patients and controls were interviewed in Ghana and Benin between November 2015 and May 2017.

#### Scale development process

In this phase, the questionnaire was tested for construct validity, inter-interviewer reliability (1-month interval), and known groups validity (discrimination between patients and controls) in Ghana and Benin.

### Construct validity

To test the ability of the PP-scale to measure the intended social construct of participation, 6 *a priori* hypotheses were formulated similar to the study by the Zeeuw et al. (2014) but adjusted for children (box 1). In total 5 out of 6 of the hypotheses need to be confirmed to positively rate construct validity [11].

Hypothesis 1: Former BU patients under 15 years with category III lesions have higher PP-scale scores than those with category I & II lesions. (In de Zeeuw et al, hypothesis number one was based on the variable ‘visible deformities’. Since this variable was not reliably scored in our study population, we replaced this hypothesis with ‘category III lesions’, being another indicator predicting long term problems).

Hypothesis 2: Former BU patients under 15 years with a joint involved have significantly higher PP-scale scores than those without.

Hypothesis 3: Former BU patients under 15 years who have stopped attending school have significantly higher PP-scale scores than those who continued attending school.

Hypothesis 4: A positive correlation (0.4 ≤ r ≤ 0.8) exists between the PP-scale sum scores and the Buruli Ulcer Functional Limitation Scores (BUFLS).

Hypothesis 5: A positive correlation (0.4 ≤ r ≤ 0.8) exists between the PP-scale sum scores and the Children’s Dermatology Life Quality Index (CDLQI) scores.

Hypothesis 6: A high level of agreement (Cohen’s κ ≥ 0.7) exists between the PP-scale sum scores of former patients with BU and their relatives [12].

### Reliability

The inter observer reliability was calculated in order to find the amount of agreement and error between different interviewers. A retest of the PP-scale within one month after first assessment was performed in Ghana by an interviewer other than the initial tester. The interclass correlation coefficient (ICC_agreement_) was used to analyse the inter-observer reliability. An ICC score of 0.7 and a minimum of 50 observations is considered as minimum to positively rate reliability [14].

### Floor and ceiling effects

Floor and ceiling effects were considered present if >15% of participants scored lowest (0) or highest (80) possible score on the PP-scale [14].

### Discrimination

To test discriminative value of the instrument, former patients and healthy controls were interviewed. Questions were classified as being discriminative if there was a significant difference (p<0.05) in PP-scale sum scores between participants and healthy controls.

### Questionnaires

The Buruli Ulcer Functional Limitation Score (BUFLS) and the Children’s Dermatology Life Quality Index (CDLQI) were used to test construct validity of the PP-scale.

**BUFLS:** The BUFLS measures functional limitations among (former) BU patients. The questionnaire covers four areas of activity and corresponds to 19 items of day-to-day activities. The four areas are preparation of food/eating (four questions), clothing/personal care taking (three questions), working (five questions), and mobility (seven questions). Responses are scored as 0‘easy/normal, 1‘with difficulties’ and 2‘not possible at all’. The total score is calculated as sum of individual scores divided by the maximal score of all applicable items, multiplied by 100. Possible values range from 0–100 percent, a score of 0 implies no functional limitations and a higher score means more functional limitations [15,16].

***CDLQI*:** The CDLQI measures the impact of skin disease on the lives of children aged from 4 to 16 years. The 10 items cover six areas of daily activities including symptoms and feelings, leisure, school or holidays, personal relationships, sleep, and treatment. The questions are based on the preceding week to minimize recall bias. Each item is answered on a 4-point Likert scale scored from 0 to 3. These are added to give a minimum score of 0 and maximum score of 30. A higher CDLQI score indicates greater degree of Quality of Life impairment [17]. The CDLQI is a well validated tool used in 28 countries worldwide, including low-income countries as Ethiopia and Ghana [24]. It was used previously in a study on the Quality of Life (QoL) among 54 former BU patients in Ghana but has not been validated in Ghana before [24].

### Procedure

Before testing the questionnaire in PHASE III, all items of the draft PP-scale were translated from English to French and backwards with the help of well-trained translators. This included thorough discussion of the interpretation of questions at the start of data collection and at several meetings during data collection. We worked with the same translators during the entire study period. Former patients were interviewed in their local language (Twi in Ghana, Fon in Benin) but this was not written down because these languages are not often written down or read. Native speakers discussed the items with the translators to ensure correct translation of the items.

To ensure privacy during the interviews, we tried to find quiet places, either at school or at home, to conduct the interviews. The other two questionnaires (BULFS, CDLQI) are available in English and French.

### Ethical consent

The Medical Ethical Review committees of the Kwame Nkrumah University of Science and Technology; School of Medical Sciences, Komfo Anokye Teaching Hospital in Ghana (ref: CHRPE/RC/127/12) and Ministry of Health in Benin (ref: No012/MS/DC/SGM/DFR7CNERS/SA) approved the study. Before each interview the procedure was explained and written informed consent was obtained from both the participants and parent or caretaker, as by definition, all participants were under the age of 15. If the participant was not able to read, the consent form was read aloud by the interpreter. If the participant was not able to write but agreed to participate, a fingerprint (thumb print) was used. No incentives were paid to the participants, only small goods such as snacks were given to the participants.

### Statistics

Data was analysed using STATA version 15.1. Descriptive statistics were used to describe baseline characteristics. Correlation of the items with sociodemographic variables was tested using Spearman’s rho. The Mann-Whitney U test was used to analyse potential differences between PP-scale scores by category of lesion, joint involvement, and socio-economic characteristics. Spearman’s rho was calculated to assess the strength of association between PP-scale score and scores of the BUFLS and CDQLI questionnaires. Cohen’s kappa and Spearman’s rho were used to compare the total PP-scale scores as reported by the participant and those reported by the accompanying relative. Rationale for use of the kappa agreement instead of another correlation test was that parents are likely to rate the importance of the included items differently. Inter observer reliability was assessed using the intraclass correlation coefficient (ICC) and Bland-Altman plot analysis of agreement (95% CI). Discrimination ability between patients and controls was tested using Mann-Whitney U.

## Results

### Phase I: Item collection and formulation of first draft questionnaire

#### Study population

For the scale development, a total of 49 persons were questioned in Benin; 7 teachers, 3 village chiefs, 1 nurse, 1 medical researcher, 4 medical assistants, 4 relatives of children with BU, 4 former adult patients suffering of BU, 18 former paediatric patients and 7 other adults.

#### Scale development process

After interviews with all informants, a list of items was composed in close collaboration with the informants. All items were checked for interpretability and content validity, resulting in 25 items that were formulated in French and English (S1 Appendix).

### Phase II: Item reduction analysis and content validity of the draft scale

#### Study population

In November 2015, 15 former BU patients younger than 15 years of age were interviewed to test the comprehensibility of the questions and the response options. The (mean(sd) age was 11.3 (± 0.57) years and 6 were male (Phase II.I).

In November and December 2015, 46 former BU patients under 15 years of age were interviewed to test endorsement, discrimination ability and sociodemographic correlation of the questionnaire. The mean (SD) age was 10.8 ((± 0.34)), 24 (52%) were male, and 26 (57%) attended primary school (Phase II.2).

#### Scale development process

*Phase II*.*1*: Response options were tested among the 15 former BU patients and minor changes were made to increase the comprehensiveness.

*Phase II*.*2*: Testing for endorsement, discrimination ability, sociodemographic correlations resulted in the elimination of 9 items. Two items (Q1 & Q17) were eliminated due to a single correlation with respectively age and the location of the lesion. Seven items were eliminated due to a very weak level of internal consistency (Q8, Q9, Q12, Q13, Q14, Q15, Q17, Q18). The final questionnaire consisted of 16 items (Q2, Q3, Q4, Q5, Q6, Q7, Q10, Q11, Q16, Q19 t/m 25) and this questionnaire was tested in PHASE III (S2 Appendix).

### Phase III: Psychometric testing and analysis

#### Study population

In Benin, 679 former paediatric BU patients in Benin were identified using medical records of the years 2005–2012. Of the 679 identified former patients, 289 had reached an age less than 15 at time of the interview and were therefore not eligible. In total 90 (31%) former paediatric BU patients were interviewed in their villages between February 2016 and May 2017 in Benin.

In Ghana, 109 former paediatric BU patients were interviewed at schools located within region of the hospital between February 2016 and May 2017. Of the total 199 participants, 113 (57%) were male and the mean(sd) age at time of data collection was 10.5 (± 0.17). Most of the former patients in Ghana had category I lesions at start of treatment and the majority of former patients in Benin had category II lesions at start of treatment. Former BU patients in Ghana had a median (IQR) PP-scale score of 0 (0–2) while former patients in Benin had a median (IQR) PP-scale score of 6 (2–14) (Table 1).

**Table 1:** Baseline characteristics of study population (Phase III).

	N = 109	N = 90	N = 199
PLACE OF INCLUSION	Ghana	Benin	Total
**AGE AT TIME OF INCLUSION (MEAN(SD))**	11.0 (2.4)	9.9 (2.4)	10.5 (0.17)
**MALE N (%)**	63 (58)	50 (40)	113 (57)
CATEGORY LESION N (%)* I **II** **III**	65 (59.6) 24 (22.0) 20 (18.4)	25 (35.7) 32 (45.7) 13 (18.6)	90 (50.3) 56 (31.3) 33 (18.4)
**JOINT INVOLVEMENT YES (%)**	26 (24)	20 (22)	46 (23)
IN SCHOOL AFTER TREATMENT YES** (%)	94 (86.2)	48 (53.3)	142(71.4)
**SCHOOL DROP-OUT DUE TO BU YES (%)**	2 (2)	12 (13)	22 (11.1)
**PP-SCALE SCORE MEDIAN(IQR) [RANGE] (%-PP-SCALE SCORE ZERO)**	0 (0;2) [0–50] (70%)	6 (2;14) [0–59] (22%)	1 (0;9) [0–59] (48%)
**BUFL SCORE (MEDIAN(IQR)**	0 (0;7.9)	0 (0;10.5)	0(0;3)
**CDLQI SCORE (MEDIAN(IQR)**	3 (0.5;7)	8 (5;10)	6(2;9)

*Category of lesion missing for 20 patients in Benin and for 8 patients in Ghana

** Information on school attendance was missing for 4 patients in Benin. Abbreviations: *BU = Buruli Ulcer; IQR = Inter Quartile Range; PP = Paediatric Participation; BUFL = Buruli Ulcer Functional Limitations; CDLQI = Children’s Dermatology Life Quality Index*

**PP-scale testing:** Construct validity, reliability, and discrimination of the draft PP-Scale were tested.

### Validity

Hypothesis 1: Former patients with category III lesions have higher PP-scale scores (n = 33, median (IQR) = 3(0;16)) than those with category I or II lesions (n = 146, median (IQR) = 0(0;6) (z = -1.79, p = 0.073).

Hypothesis 2: Former patients with a joint involved have higher PP-scale scores (n = 46, median (IQR) = 1(0;11)) than those without (n = 153, median (IQR) = 0(0;7) (z = -0.86, p = 0.39)).

Hypothesis 3: In Benin, the PP-scale score of patients who dropped out from school after treatment due to BU (n = 12, median (IQR) 9(5;18)) is higher than the score of children who attended school after treatment (n = 48, median (IQR) 3(0;12) (z = -1.82; p = 0.070)).

Hypothesis 4: A positive correlation of 0.552 (Spearman, n = 198, p< 0.001) exists between the PP-scale sum scores and the BUFLS sum scores. The BUFLS had a median (IQR) score of 0 (0;7.9) in Benin and 0 (0;10.5) in Ghana. The BUFLS was more than 0 (indicating a functional limitation) in 47.2% (n = 42) in Benin and 39.5% (n = 43) in Ghana.

Hypothesis 5: A positive correlation of 0.638 (Spearman, n = 196, p< 0.001) was found between the PP-scale sum scores and the CDLQI scores. The CDQLI had a median (IQR) of 8 (5;10) in Benin and 3 (0.5;7) in Ghana;

Hypothesis 6: A Cohen kappa score of 0.248 (N = 62, 95% CI 0.20;0.55) patients and their relatives was found. A weak correlation of 0.343 (Spearman, n = 62, P<0.01)) was found between the PP-scale sum scores of former patients with BU and the score as reported by their relatives (S3 Appendix).

### Inter-observer reliability

To test inter-observer reliability, the scores of 32 participants were reassessed. An ICC score of 0.379 (95% CI (0.04;0.65)) was found. This number of observations is too small to positively rate inter-observer reliability (S3 Appendix–Bland-Altman plot: mean difference = 2.5 (95% CI (-1.2;6.3); sd of difference: 10.6, limits of agreement (reference range for difference): [-18.7;23.8]. The smallest detectable change (SDC) based on the 32 measurements for reliability analysis is 21.1 [14]. This value is larger than the considered minimal important change (MIC) and further validation with a sufficient sample size is needed.

### Floor and ceiling effects

Floor effects were present. In Benin, 22% (n = 20) of the participants scored 0 on the PP-scale and in Ghana 71% (n = 77) (X2 = 46.25, p < 0.001). None of the participants had a maximum score of 80 points, therefore no ceiling effects were present.

### Discrimination

To test for discriminative value of the instrument, 79 healthy controls were interviewed and the responses were compared with patient responses. Age and gender did not differ significantly between former BU patients and controls. Former patients had a higher median (IQR) PP-score (1(1;9)) than healthy controls (0(0;0)) (Wilcoxon rank sum z = 6.96, p < 0.0001).

### Participation restrictions

Former BU patients from Benin reported more participation restrictions compared to former patients from Ghana. In both countries, most of the reported problems were related to sports, mainly in playing games with others (Q12), going to the playfield (Q10) and doing sports at school (Q13). In Benin, former patients experienced also problems with participation in domestic life and religious ceremonies, while in Ghana this was very rare. S4 Appendix summarizes the scores per question (S4 Appendix).

In Benin, 44% (38/86) of the patients interviewed did not attend school, 32% (12/38) of whom stopped attending school because of BU. In Ghana, 14% (15/108) did not go to school, of whom 13.3% (2/15) dropped out of school because of BU. Other reasons for not attending school were poverty, lack of motivation to go to school, or other unknown reasons.

## Discussion

The aim of this study was to develop and test a scale that measures participation restrictions among former Buruli Ulcer patients under the age of 15. We developed a scale with feasible items that measures the concept of participation among children under the age of 15 but requires further testing on the validity and applicability of the scale.

We faced several constrains when testing the validity of the instrument we developed. In total, 5 out of 6 a priori set hypotheses could be confirmed but only 2 showed statistically significant associations (p <0.05). The results were in the direction as predefined, but due to a low percentage of patients with severe lesions the study lacked statistical power. A strong positive correlation was found between the PP-scale, the Buruli Ulcer Functional Limitation Score (BUFLS) and the Children’s Dermatology Life Quality Index (CDLQI) confirming construct validity. Although discriminative power of the scale was highly significant, the median difference of 1-point sum score is small and raises questions about the clinical relevance of this difference.

Participants with category III lesions or with joints involved scored slightly higher on the PP-scale. These differences suggest that physical limitations limit social participation. However, the difference related to joint involvement was small and statistically non-significant. We cannot exclude this difference to have arisen by chance. It is also possible that the scale is not sensitive in measuring smaller barriers related to physical limitations. The study by the Zeeuw et al. on the psychometric properties of the P-scale among adults found higher participation restrictions among patients with visible deformities and/or joint involvement [12].

While most patients in Ghana went to school, in Benin only 56% went to school after treatment. Reasons for drop-out were directly related to their disease such as functional limitation or stigma, or the result of indirect consequences such as expectations of (financial) compensation for their absence. However, it is difficult differentiate between indirect consequences of BU and more general socio-economic circumstances as poverty or other responsibilities.

In total, scores of 32 participants were reassessed. Based on these reliability measures, the smallest detectable change (SDC) is much larger than the minimal important change (MIC). The a priori set level of 50 participants as minimum to test validity could not be met and this should be further analysed by follow-up studies on the PP-scale.

The individual test scores are in line with previously reported problems among BU patients [8,9]. Among children, school-drop out is the major problem, especially in Benin. Other major problems are related to sports, ability to attend religious ceremonies and having friends (S4 Appendix). Former BU patients in Benin score substantially higher on most of the items of the PP-scale compared to the former patients in Ghana. The observed differences in participation restrictions, mostly related to sports and social activities, could be related to differences in socio-economic conditions and treatment outcomes (i.e. severity of lesions) between Ghana and Benin. Sociocultural differences may also have led to a different pattern in answering. Patients in Benin on average have more severe lesions than patients in Ghana, which makes it more likely that the detected differences actually reflect differences in disease outcome.

The instrument we developed was well-received by the participants and their guardians. Most of the participants were able to answer all the questions and they did not report questions being too sensitive. The procedure took approximately 20 minutes and most participants were able to pay attention until the end. Among younger participants, parents were often involved in answering the questions.

Measuring participation restrictions in children is challenging and studies on participation in children with disabilities in low-income settings are scant. Social participation among children is influenced by different factors, including socio-economic status, physical abilities, and community perception of the disease and related stigma. A study from southern Ethiopia in children with scabies and tungiasis reports a moderate impact of the disease on their quality of life, especially the ability to go to school due to physical barriers [18]. A study in South Africa and Malawi describes absence of school due to HIV related stigma and poor living conditions as major determinants of participation restrictions among HIV infected children [19].

In contrast, a study on quality of life in adults with leprosy shows that the incapacity to contribute to the family finances–and not the physical impairments—is a major driver of disease related stigma [20]. It is possible that former paediatric BU patients in this study face similar problems as a result of the indirect barriers (e.g. inability to contribute financially) related to the disease.

This study has several limitations. A major limitation was the limited ability to test validity. The defined hypotheses were based on the underlying assumption that most of the patients had moderate to severe (category II/III) lesions. However, due to the improvements in diagnosis and treatment, most of the patients in Ghana had category I lesions. This resulted in weaker associations based on the predefined hypothesis. Correspondingly, we found a floor effect where 70% of the former patients in Ghana scored 0 on the PP-scale. Therefore, to reach statistically significant results with regard to the a priori set hypotheses, a bigger sample size (or a sample of patients with more severe forms of the disease) would be needed or hypotheses should be changed based on more mild forms of BU. The low score of participation restrictions among patients from Ghana correspondents with findings of a good quality of life among former BU patients with small lesions, as a result of improvements in early identification and treatment of BU patients in Ghana [21].

The interview setting, which was unusual for many participants, may have influenced the answers of the participants. In many cases parents or other family members were involved in answering the questions, especially among the younger children, which could have influenced the answers of the participants. A relatively small proportion of the study participants were aged between 5–8 years, due to both the study design (late follow-up compared to patient identification) and the lower prevalence of BU among the younger children. Finally, the questions were formulated in French and translated to either Twi or Fon on spot, which could result in variations in translation. However, during data collection regular meetings with the translators and local health care professionals involved in the study were organized to discuss the process of the interviews, including the importance of continuing to use the exact same wording during the interviews. The same translators were used during the entire study period. Despite these limitations, this is the first research that provides an indication that former BU patients under 15-years of age may face participation restrictions in important aspects of their lives.

Participation restrictions that former patients face limits future possibilities and reduces quality of life of the individual patient. Early detection of cases and information about the disease is important to prevent social exclusion. As seen in Ghana, early case detection and improvement of treatment has large impact on improving the quality of life and participation of former patients, and this is the major factor that reduces participation restrictions. In addition, programs to improve participation of former patients in the community should be considered, similar to the existing programs for leprosy and HIV. These programs have proven to reduce stigma and social exclusion among leprosy patients [22,23]. Increasing our knowledge about participation problems may improve participation opportunities among former Buruli Ulcer paediatric patients. An instrument such as the PP-scale, when further tested and validated, could be a useful tool to identify major barriers former BU patients face, and may help local health service providers to better meet the needs of the patients.

The preliminary results of the PP-scale validation are promising. The instrument may well be valid to measure participation restrictions among children with more severe forms of BU, but future additional validation studies need to confirm this.

## Supporting information

S1 Appendix Draft questionnaire as formulated after phase I. (DOCX)

S2 Appendix Final questionnaire after elimination of 9-items (16 questions). (DOCX)

S3 Appendix Graphical presentations of test results. (DOCX)

S4 Appendix Percentages test scores PP-scale in Ghana and Benin (Phase III). (DOCX)
