# Supplementary material for: The paediatric participation scale measuring participation restrictions among former Buruli Ulcer patients under the age of 15 in Ghana and Benin: Development and first validation results
Source: PLoS Negl Trop Dis. 2019 Mar 14;13(3):e0007273. doi: 10.1371/journal.pntd.0007273 (PMC6435175; doi:10.1371/journal.pntd.0007273)
Supplement: S1 Appendix — (DOCX) [file pntd.0007273.s001.docx]

**S1 Appendix. Draft questionnaire as formulated after phase I.**

|  | Paediatric Participation Scale (PP-Scale) | **Not Specified, not answered** | **Yes** | **Sometimes** | **No** | **Irrelevant, I don’t want to , I don’t have to** | **Inappropiate** | **No problem** | **A little bit important / a little bit difficult** | **Medium important / medium difficult** | **Important / too difficult** | **TOTAL** |
| --- | --- | --- | --- | --- | --- | --- | --- | --- | --- | --- | --- | --- |
| Q1 | Do you move around inside and outside your house just as other children do? | 8 | 0 |  |  | 9 | 10 | 1 | 2 | 3 | 5 |  |
| **Self care** | | | | | | | | |  |  |  |  |
| **Q2** | **Do you have the opportunity to take care of yourself just like the other children do? Ex) to wash yourself, to brush your teeth, to go to the bathroom, to dress, to eat and drink** | **8** | **0** |  |  | **9** | **10** | **1** | **2** | **3** | **5** |  |
| **Domestic tasks** | | | | | | | | | | |  |  |
| **Q3** | **Can you go to the market just like the other children do?** | **8** | **0** |  |  | **9** | **10** | **1** | **2** | **3** | **5** |  |
| **Q4** | **Can you help your parents to prepare dinner just like the other children do?** | **8** | **0** |  |  | **9** | **10** | **1** | **2** | **3** | **5** |  |
| Q5 | In your home, do you do household work just like the other children do? Ex) sweep the yard of the house, clean the house, do the dishes, wash the clothes | 8 | 0 |  |  | 9 | 10 | 1 | 2 | 3 | 5 |  |
| **Q6** | **Do you help other people just like the other children do? ex) neighbours, friends or people in your community** | **8** | **0** |  |  | **9** | **10** | **1** | **2** | **3** | **5** |  |
| **Q7** | **Do you go to get water yourself just like the other children do?** | **8** | **0** |  |  | **9** | **10** | **1** | **2** | **3** | **5** |  |
| **Interpersonal interactions and relationships** | | | | | | | | | | |  |  |
| Q8 | Are you respected by the other children just like they respect children who haven’t suffered from BU? | 8 | 0 |  |  | 9 | 10 | 1 | 2 | 3 | 5 |  |
| Q9 | Are you comfortable meeting new people just like the other children are? Ex) classmates, neighbours, adults | 8 | 0 |  |  | 9 | 10 | 1 | 2 | 3 | 5 |  |
| **Q10** | **Do you have as many friends as children that haven’t suffered from BU?** | **8** | **0** |  |  | **9** | **10** | **1** | **2** | **3** | **5** |  |
| **Q11** | **Do you have good relationships with your siblings just like they understand each other?** | **8** | **0** |  |  | **9** | **10** | **1** | **2** | **3** | **5** |  |
| Q12 | Do you go to the same school as the children that haven’t had BU? | 8 | 0 |  |  | 9 | 10 | 1 | 2 | 3 | 5 |  |
| Q13 | Do you have the same opportunity as the other children to enter secondary school | 8 | 0 |  |  | 9 | 10 | 1 | 2 | 3 | 5 |  |
| Q14 | Do you play with the other kids (during the school break) like they are playing with each other? | 8 | 0 |  |  | 9 | 10 | 1 | 2 | 3 | 5 |  |
| Q15 | Do you play with the other kids (during the school break) like they are playing with each other? | 8 | 0 |  |  | 9 | 10 | 1 | 2 | 3 | 5 |  |
| **Community, social and civic life** | | | | | | | | | | | | |
| **Q16** | **Do you attend ceremonies and family parties as much as the other children? Ex) Marriage, funerals?** | 8 | 0 |  |  | 9 | 10 | 1 | 2 | 3 | 5 |  |
| Q17 | Do you help your parents preparing attend ceremonies and family parties as much as the other children do? Ex) Marriage, funerals? | 8 | 0 |  |  | 9 | 10 | 1 | 2 | 3 | 5 |  |
| Q18 | Do you have the same responsibilities during ceremonies and family parties to as the other children? Ex: to sing, to 0dance to play music | 8 | 0 |  |  | 9 | 10 | 1 | 2 | 3 | 5 |  |
| **Q19** | **Do you often go to the field to play with the other kids just like the other children do? ex) ball games** | **8** | **0** |  |  | **9** | **10** | **1** | **2** | **3** | **5** |  |
| **Q20** | **Do you often go to your neighbours to play with the other kids just like the other children do?** | **8** | **0** |  |  | **9** | **10** | **1** | **2** | **3** | **5** |  |
| **Q21** | **Do you play the same games as the other children during your free time? Ex: football** | **8** | **0** |  |  | **9** | **10** | **1** | **2** | **3** | **5** |  |
| **Q22** | **Do you sport at school in the same way that the other children do?** | **8** | **0** |  |  | **9** | **10** | **1** | **2** | **3** | **5** |  |
| **Q23** | **Do you often visit your friends who live far from home just like the other children do?** | **8** | **0** |  |  | **9** | **10** | **1** | **2** | **3** | **5** |  |
| **Q24** | **Do you go to church, mosque or other religious place as much as the other children?** | **8** | **0** |  |  | **9** | **10** | **1** | **2** | **3** | **5** |  |
| **Q25** | **Do you have the same responsibilities in the church, mosque or other religious place as the other children do? Ex) sing, dance.** | **8** | **0** |  |  | **9** | **10** | **1** | **2** | **3** | **5** |  |
