# Supplementary material for: The paediatric participation scale measuring participation restrictions among former Buruli Ulcer patients under the age of 15 in Ghana and Benin: Development and first validation results
Source: PLoS Negl Trop Dis. 2019 Mar 14;13(3):e0007273. doi: 10.1371/journal.pntd.0007273 (PMC6435175; doi:10.1371/journal.pntd.0007273)
Supplement: S2 Appendix — (DOCX) [file pntd.0007273.s002.docx]

**Paediatric Participation Scale (PP-scale) V.1**

|  | Paediatric Participation Scale (PP-scale) V.1 | Not specified, not  answered | Yes | Sometimes | No | Irrelevant, I don’t  want to, don’t have to | No problem | A little bit important | Important | Very important | SCORE |
| --- | --- | --- | --- | --- | --- | --- | --- | --- | --- | --- | --- |
| 1 | Do you have the opportunity to take care of yourself just like the other children do? Ex) to wash yourself, to brush your teeth, to go to the bathroom, to dress, to eat and drink |  | 0 |  |  | 0 |  |  |  |  |  |
|  | [if sometimes or no] How big a problem is it to you? |  |  |  |  |  | 1 | 2 | 3 | 5 |  |
| 2 | Can you go to the market just like the other children do? |  | 0 |  |  | 0 |  |  |  |  |  |
|  | [if sometimes or no] How big a problem is it to you? |  |  |  |  |  | 1 | 2 | 3 | 5 |  |
| 3 | Can you help your parents to prepare dinner just like the other children do? |  | 0 |  |  | 0 |  |  |  |  |  |
|  | [if sometimes or no] How big a problem is it to you? |  |  |  |  |  | 1 | 2 | 3 | 5 |  |
| 4 | In your home, do you do household work just like the other children do? Ex) sweep the yard of the house, clean the house, do the dishes, wash the clothes |  | 0 |  |  | 0 |  |  |  |  |  |
|  | [if sometimes or no] How big a problem is it to you? |  |  |  |  |  | 1 | 2 | 3 | 5 |  |
| 5 | Do you help other people just like the other children do? ex) neighbours, friends or people in your community |  | 0 |  |  | 0 |  |  |  |  |  |
|  | [if sometimes or no] How big a problem is it to you? |  |  |  |  |  | 1 | 2 | 3 | 5 |  |
| 6 | Do you go to get water yourself just like the other children do? |  | 0 |  |  | 0 |  |  |  |  |  |
|  | [if sometimes or no] How big a problem is it to you? |  |  |  |  |  | 1 | 2 | 3 | 5 |  |
| 7 | Do you have as many friends as children that haven’t suffered from BU? |  | 0 |  |  | 0 |  |  |  |  |  |
|  | [if sometimes or no] How big a problem is it to you? |  |  |  |  |  | 1 | 2 | 3 | 5 |  |
| 8 | Do you have good relationships with your siblings just like they understand each other? |  | 0 |  |  | 0 |  |  |  |  |  |
|  | [if sometimes or no] How big a problem is it to you? |  |  |  |  |  | 1 | 2 | 3 | 5 |  |
| 9 | Do you attend ceremonies and family parties as much as the other children? Ex) Marriage, funerals? |  | 0 |  |  | 0 |  |  |  |  |  |
|  | [if sometimes or no] How big a problem is it to you? |  |  |  |  |  | 1 | 2 | 3 | 5 |  |
| 10 | Do you often go to the field to play with the other kids just like the other children do? ex) ball games |  | 0 |  |  | 0 |  |  |  |  |  |
|  | [if sometimes or no] How big a problem is it to you? |  |  |  |  |  | 1 | 2 | 3 | 5 |  |
| 11 | Do you often go to your neighbours to play with the other kids just like the other children do? |  | 0 |  |  | 0 |  |  |  |  |  |
|  | [if sometimes or no] How big a problem is it for you? |  |  |  |  |  | 1 | 2 | 3 | 5 |  |
| 12 | Do you play the same games as the other children during your free time? Ex: football |  | 0 |  |  | 0 |  |  |  |  |  |
|  | [if sometimes or no] How big a problem is it to you? |  |  |  |  |  | 1 | 2 | 3 | 5 |  |
| 13 | Do you sport at school in the same way that the other children do? |  | 0 |  |  | 0 |  |  |  |  |  |
|  | [if sometimes or no] How big a problem is it to you? |  |  |  |  |  | 1 | 2 | 3 | 5 |  |
| 14 | Do you often visit your friends who live far from home just like the other children do? |  | 0 |  |  | 0 |  |  |  |  |  |
|  | [if sometimes or no] How big a problem is it to you? |  |  |  |  |  | 1 | 2 | 3 | 5 |  |
| 15 | Do you go to church, mosque or other religious place as much as the other children? |  | 0 |  |  | 0 |  |  |  |  |  |
|  | [if sometimes or no] How big a problem is it to you? |  |  |  |  |  | 1 | 2 | 3 | 5 |  |
| 16 | Do you have the same responsibilities in the church, mosque or other religious place as the other children do? Ex) sing, dance. |  | 0 |  |  | 0 |  |  |  |  |  |
|  | [if sometimes or no] How big a problem is it to you? |  |  |  |  |  | 1 | 2 | 3 | 5 |  |

*The total sum-score can be calculated by summing up all the individual items.

**TOTAL**

Comment:

Name: _________________________________________

Age: ____ Gender: ______

Interviewer: ___________________________________ Date of interview: ___ / ___ / ____

**Disclaimer: This is a preliminary version of the Paediatric Participation Scale and further validation is required before use. Contact the authors if you are interested in using the scale. The authors who developed the scale and the development team cannot be held responsible for any consequences of the use of the Paediatric Participation Scale.**
