# Supplementary material for: The paediatric participation scale measuring participation restrictions among former Buruli Ulcer patients under the age of 15 in Ghana and Benin: Development and first validation results
Source: PLoS Negl Trop Dis. 2019 Mar 14;13(3):e0007273. doi: 10.1371/journal.pntd.0007273 (PMC6435175; doi:10.1371/journal.pntd.0007273)
Supplement: S3 Appendix — (DOCX) [file pntd.0007273.s003.docx]

**S3 Appendix.

Fig S1. Bland-Altman plot to test inter-observer reliability.**


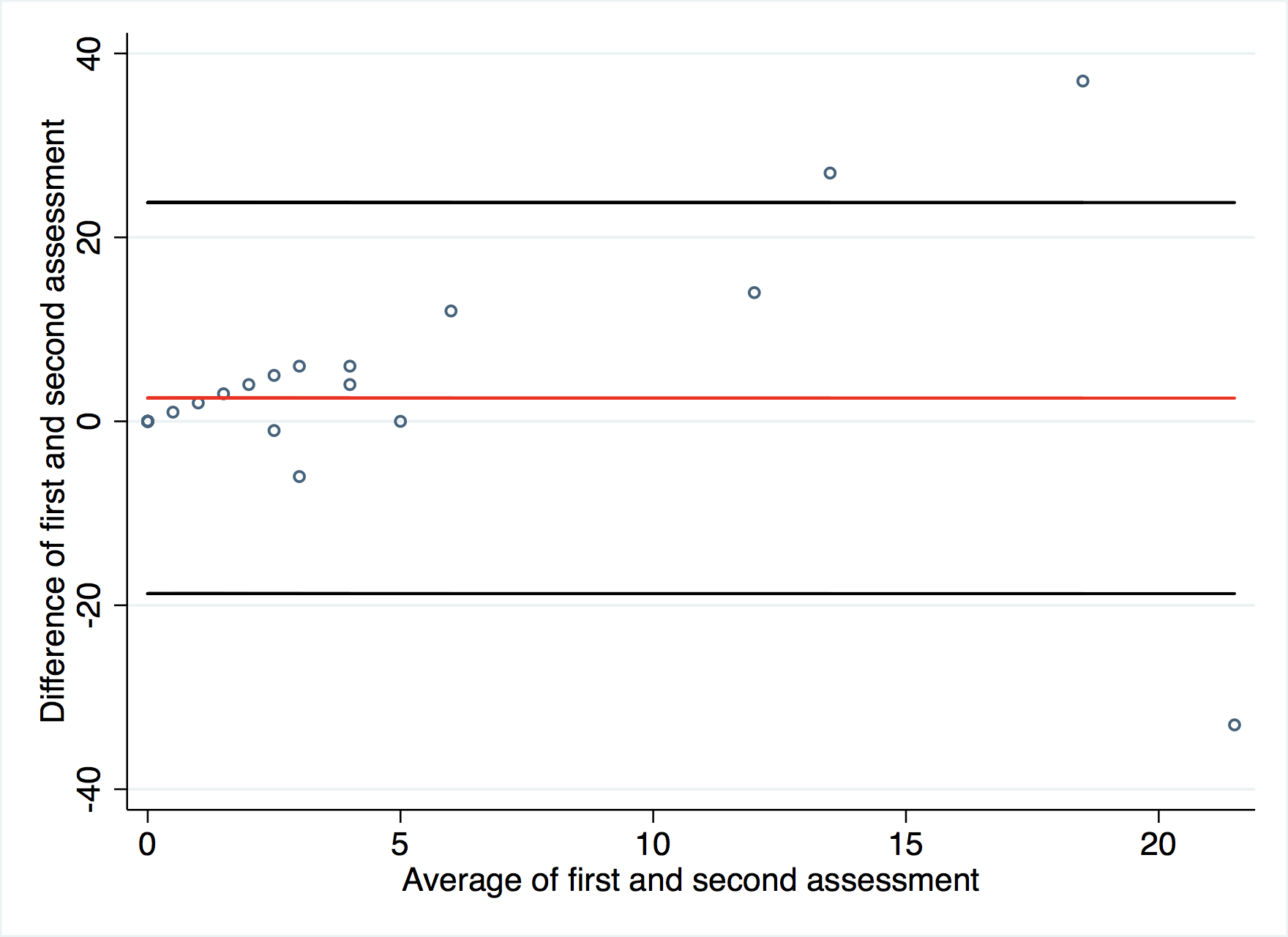


Bland-Altman plot to assess inter-observer reliability: agreement of PP-scale scores of participants between first and second assessment (n=26). Mean difference = 2.5 (95% CI (-1.2;6.3); sd of difference: 10.6, Limits of agreement (Reference range for difference): -18.7 – 23.8.

**Fig S2. Graphical presentations of the hypothesis tested as part of construct validity**

(a) (b)

(c) (d) (e)_ (f)
Fig 2a-c: Boxplot (Median (IQR), outside values) of PP-scale sum scores and Wilcoxon rank-sum test results for (a) hypothesis 1: Category of lesion; (b) hypothesis 2: Joint involvement; (c) hypothesis 3: School attendance.
Fig 2d-f: Scatter plot PP-scale sum scores and spearman rank test results for (d) hypothesis 4: BUFLS scores; (e) hypothesis 5: CDLQI scores; (f) hypothesis 6: PP-scale-score by relatives.
* Abbreviations: *BU = Buruli Ulcer; IQR = Inter Quartile Range; PP=Pediatric Participation; BUFL= Buruli Ulcer Functional Limitations; CDLQI = Children’s Dermatology Life Quality Index*
