# Supplementary material for: The paediatric participation scale measuring participation restrictions among former Buruli Ulcer patients under the age of 15 in Ghana and Benin: Development and first validation results
Source: PLoS Negl Trop Dis. 2019 Mar 14;13(3):e0007273. doi: 10.1371/journal.pntd.0007273 (PMC6435175; doi:10.1371/journal.pntd.0007273)
Supplement: S4 Appendix — (DOCX) [file pntd.0007273.s004.docx]

**S4 Appendix. Percentages test scores PP-scale in Ghana and Benin (Phase III).**

| Domain of participation restriction | Question | Ghana N=109 % | | | | | Benin (N=90) % | | | | |
| --- | --- | --- | --- | --- | --- | --- | --- | --- | --- | --- | --- |
|  |  | **Not restricted** | **No problem** | **Small problem** | **Medium problem** | **Large problem** | **Not restricted** | **No problem** | **Small problem** | **Medium problem** | **Large problem** |
| Self-care | 1. Personal care | 97.2 | - | - | 0.9 | 1.8 | 92.2 | - | 3.3 | 3.3 | 1.1 |
| Domestic life | 2. Market | 96.3 | - | 0-9 | 0.9 | 1.8 | 77.8 | 2.2 | 8.9 | 7.8 | 3.3 |
|  | 3. Help with diner preparation | 94.5 | - | 2.8 | - | 2.8 | 83.3 | 2.2 | 6.7 | 3.3 | 4.4 |
|  | 4. Work in household | 90.8 | 2.8 | 2.8 | - | 3.7 | 77.8 | 1.1 | 7.8 | 6.7 | 6.7 |
|  | 5. Help other people | 92.7 | 0.9 | 1.8 | 1.8 | 2.8 | 84.4 | - | 6.7 | 5.6 | 3.3 |
|  | 6. Get water | 94.5 | - | 0.9 | - | 4.6 | 86.7 | 1.1 | 2.2 | 7.8 | 2.2 |
| Interpersonal interactions and relationships | 7. As many friends as other children | 95.4 | 1.8 | 00 | - | 2.8 | 74.4 | - | 8.9 | 12.2 | 4.4 |
|  | 8. Relationship with siblings | 99.1 | - | - | - | 0.9 | 84.4 | 1.1 | 5.6 | 6.7 | 2.2 |
| Community. Social. And civic life | 9. Attend ceremonies | 94.5 | 0.9 | - | - | 4.6 | 65.6 | 1.1 | 7.8 | 13.3 | 12.2 |
|  | 10. Go to the playfield | 88.1 | 2.8 | 1.8 | 1.8 | 5.5 | 63.3 | - | 6.7 | 15.6 | 14.4 |
|  | 11. visit neighbours | 95.4 | - | - | - | 4.6 | 73.3 | - | 4.4 | 11.1 | 11.1 |
|  | 12. play similar games | 84.4 | 0.9 | 1.8 | 0.9 | 11.9 | 78.9 | 1.1 | 1- | 6.7 | 3.3 |
|  | 13. Sports at school | 87.2 | 0.9 | 2.8 | 1.8 | 11.9 | 47.8 | - | - | 4.4 | 5.6 |
|  | 14. Visit friends far from home | 89.0 | - | 1.8 | 0.9 | 8.3 | 86.7 | 1.1 | 4.4 | 4.4 | 3.3 |
|  | 15. Visit religious ceremony | 99.1 | - | - | - | 0.9 | 97.8 | - | - | 1.1 | 1.1 |
|  | 16. Involvement religious responsibilities | 92.7 | 1.8 | 0.9 | - | 4.6 | 98.9 | - | - | - | 1.1 |
